# Breast milk Δ9-tetrahydrocannabinol in cannabis users during the postpartum period: correlation between breast milk, maternal urine and saliva samples during early lactation

**DOI:** 10.3389/fpsyt.2026.1816611

**Published:** 2026-06-30

**Authors:** Miguel Sandonís, Maia Brik, Clara Ramirez, Laura Castellote, Anna Creus, Gemma Parramón, Anna Suy, Josep Antoni Ramos-Quiroga, Raul Felipe Palma-Álvarez, Constanza Daigre

**Affiliations:** 1 Department of Mental Health, Hospital Universitari Vall d’Hebron, Barcelona, Spain; 2 Psychiatry, Mental Health and Addictions Group, Vall d’Hebron Research Institute (VHIR), Instituto de Investigación Sanitaria Hospital Universitari Vall d’Hebron (IIS IR-HUVH), Barcelona, Catalonia, Spain; 3 Biomedical Research Networking Center in Mental Health (CIBERSAM), Barcelona, Spain; 4 Department of Psychiatry and Forensic Medicine, Universitat Autònoma de Barcelona, Barcelona, Spain; 5 Department of Obstetrics and Gynecology, Universitat Autònoma de Barcelona, Barcelona, Spain; 6 Obstetrics Department, Maternal-Fetal Medicine Unit, Hospital Universitari Vall d’Hebron, Barcelona, Spain; 7 Clinical Biochemistry Research Group, Vall d’Hebron Research Institute (VHIR), Biochemical Core Facilities, Instituto de Investigación Sanitaria Hospital Universitari Vall d’Hebron (IIS IR-HUVH), Barcelona, Catalonia, Spain; 8 Department of Neonatology, Hospital Universitari Vall d’Hebron, Barcelona, Spain

**Keywords:** cannabis, pregnancy, breastfeeding, postpartum, perinatal

## Abstract

**Introduction:**

Cannabis use during pregnancy and the postpartum period has increased in recent years, raising clinical concerns regarding maternal and infant health, particularly during lactation. However, evidence regarding Δ9-THC concentrations in breast milk during the early postpartum period and their relationship with other biological matrices remains limited.

**Objective:**

This study aimed to assess Δ9-THC concentrations in breast milk and saliva, and 11-nor-9-carboxy-Δ9-tetrahydrocannabinol (THC-COOH) concentrations in urine, among postpartum women with cannabis use at the time of delivery. A secondary objective was to explore correlations between these biological matrices during early lactation.

**Methods:**

A longitudinal observational study was conducted at Vall d’Hebron University Hospital (Barcelona, Spain) between April 2022 and December 2023. Thirteen postpartum women aged over 18 years with a positive urine test for cannabis at delivery and intent to breastfeed were included. Saliva, urine, and breast milk samples were collected at 24 hours, 48 hours, and one week after birth. Δ9-THC concentrations in breast milk and saliva and THC-COOH concentrations in urine were analyzed using liquid chromatography–tandem mass spectrometry (LC-MS/MS).

**Results:**

Among participants who remained abstinent during the first postpartum week, urinary THC-COOH concentrations progressively decreased but remained quantifiable across all study stages. In contrast, Δ9-THC concentrations in breast milk decreased over time and were below the limit of quantification (LOQ) one week postpartum. Salivary Δ9-THC concentrations were generally low and frequently below the LOQ. Breast milk Δ9-THC concentrations at the first sampling stage were significantly correlated with salivary Δ9-THC and urinary THC-COOH concentrations, whereas no significant correlations were observed at later stages.

**Conclusions:**

This preliminary study suggests that Δ9-THC concentrations in breast milk may decline rapidly after postpartum cannabis cessation, becoming non-quantifiable within the first postpartum week among participants who discontinued use after delivery. In contrast, urinary THC-COOH remained quantifiable for a longer period. Salivary Δ9-THC showed limited concordance with breast milk Δ9-THC and should therefore be interpreted cautiously as a potential surrogate marker. Larger prospective studies are needed to confirm these findings and to support evidence-based breastfeeding counseling for women with recent cannabis use.

## 1 Introduction

Cannabis is the third most used substance worldwide, and its use has increased substantially over the past decade, particularly among young adults, probably due to a decreased perception of risk and easy accessibility (1). In the United States, the prevalence of cannabis use was estimated at 18.7% in 2021, with the highest rates reported among individuals aged 18–25 years, reaching 35.4% (2). Cannabis use among women of reproductive age and pregnant women has increased significantly, becoming a therapeutic and public health challenge. Several factors may explain this trend (3–5). First, similarly to the general population, pregnant women may perceive cannabis use as low risk (5). Second, cannabis use during pregnancy may be related to self-treatment of several symptoms such as nausea, emesis, anxiety, insomnia, pain, among others (3, 4, 6). In addition, stigma surrounding substance use during pregnancy may negatively affect treatment-seeking and access to healthcare services (7).

Studying and accurately determining the real prevalence of cannabis use during the perinatal period remains highly challenging, since stigma and fear of legal or social consequences associated with cannabis use during pregnancy or breastfeeding may exist (7, 8). Large datasets from the United States have reported that cannabis use among pregnant women ranges between 2.3% to 6.7% (4, 7, 9). In addition, important differences in cannabis use have been observed according to maternal age, with higher prevalence among pregnant adolescents compared with adult pregnant women (9) as well as according to the reproductive period, with higher prevalence during the periconceptional and postpartum periods than during pregnancy itself (4). Most available data have been obtained through self-reporting rather than biological testing, which represents an important limitation in accurately determining true cannabis use as has been demonstrated in previous studies with discrepancies between self-reporting cannabis use and biological testing (10–12).

Breastfeeding is strongly recommended by the World Health Organization (WHO) (13) and other organizations (14). However, cannabis use may increase during the postpartum period among women who reduced or discontinued use during pregnancy (4). Several studies report that the majority of women who use cannabis during breastfeeding do so because they have not received information from healthcare professionals regarding the potential risks associated with its use (5) and many of them reported using cannabis for mental or physical health conditions (15). Consequently, important concerns have emerged regarding breastfeeding among women who use cannabis. Currently, due to the limited evidence available in this field, there are no general recommendations regarding cannabis use during breastfeeding beyond abstinence (16). Furthermore, the presence of cannabinoids in mammary adipose tissue may interfere with the lactogenesis process through direct activation of cannabinoid receptor, potentially leading to changes in breast milk composition, particularly lipid and fatty acid profiles (17). However, despite recommendations discouraging cannabis use, breastfeeding itself should not be contraindicated, given the well-documented benefits for both the infant and the lactating individual (18).

Regarding pharmacokinetic studies, cannabis contains multiple compounds; however, Δ9-THC and cannabidiol (CBD) are the most clinically relevant (19). Specifically, Δ9-THC has been associated with psychopathological alterations in cognition, emotion, and perception. Consequently, cannabis is considered a risk factor for psychosis, addiction, and other neuropsychiatric disorders (1, 8, 20). Screening for cannabis use usually involves the measurement of Δ9-THC or its metabolites (8, 21). Δ9-THC is highly lipophilic (22, 23), which explains why it may be detected for prolonged periods after cannabis use (21).

With regard to research on Δ9-THC in breast milk, one study conducted on women who had abstained from cannabis for at least 24 hours found a peak Δ9-THC concentration in breast milk one hour after controlled use (following maternal inhalation of 0.1 g of cannabis containing 23.18% Δ9-THC), which gradually decreased over the subsequent 4 hours (24). In *The Lactation and Cannabis (LAC) Study*, a prospective clinical study involving 20 breastfeeding individuals with frequent cannabis use, the estimated half-life of Δ9-THC in breast milk was 11.45 hours, with the highest Δ9-THC concentration observed in milk samples collected 120–135 minutes after cannabis use (25). Another research involving regular cannabis users found that Δ9-THC was quantifiable in 63% of milk samples up to 6 days after the participants’ last reported use (26).

When analyzing Δ9-THC levels in biological samples other than breast milk, pharmacokinetic studies suggest that saliva is a highly useful for detecting recent use of cannabis (8). The presence of Δ9-THC in oral mucosa represents recent use, as Δ9-THC may appear either through direct contact following cannabis use or through passive diffusion after peak plasma THC concentrations (21). In contrast, urinary THC metabolite levels (THC-COOH) appear to be more relevant for assessing past exposure, since THC-COOH may remain quantifiable for several weeks after use (8).

To our knowledge, there is a paucity of literature regarding the correlation between Δ9-THC levels in breast milk and other biological fluids, particularly when assessed using non-invasive techniques. According to a limited number of studies conducted in postpartum women, there is a correlation between plasma Δ9-THC and breast milk Δ9-THC (27), with higher Δ9-THC concentrations in breast milk than in plasma, showing a milk-to-plasma (M/P) ratio of approximately 8:1 (28). This study aimed to analyze Δ9-THC concentrations in saliva and breast milk at different postpartum time points and compare them with urinary THC-COOH concentrations to explore possible correlations between biological matrices.

## 2 Materials and methods

### 2.1 Participants and study design

This longitudinal observational study was conducted at the Perinatal Mental Health Service of Vall d’Hebron University Hospital between April 1, 2022, and December 31, 2023. The Perinatal Mental Health Service specializes in the care of women with mental health conditions during the preconception period, pregnancy, and postpartum (up to one year after childbirth). The unit comprises a multidisciplinary team of professionals in psychiatry, psychology, gynecology and obstetrics, nursing, and social work. Vall d’Hebron University Hospital serves a population of approximately 600,000 people in the city of Barcelona (Spain) and attends around 3,000 births annually.

In accordance with established local clinical protocols, all pregnant women were systematically screened for substance use using the World Health Organization Alcohol, Smoking and Substance Involvement Screening Test (WHO ASSIST). Women with a positive screening result subsequently underwent urine toxicology testing for 11-nor-9-carboxy-Δ9-tetrahydrocannabinol (THC-COOH) at the time of delivery using a semiquantitative technique with a cutoff value established at 50 ng/mL, after providing oral informed consent, which was documented in their medical records.

Women with a positive urine result for cannabis at the time of delivery were referred for evaluation by the liaison psychiatry team to assess substance use and were subsequently considered eligible for potential inclusion in the study.

Newborns of mothers with a positive urine test underwent qualitative urine toxicology screening for cannabis exposure using the same technique.

Patients who met the inclusion criteria and none of the exclusion criteria were invited to participate in the study. Inclusion criteria were postpartum women older than 18 years of age with a positive qualitative urine test for cannabis at the hospital admission for birth, and a self-reported intention to breastfeed. Exclusion criteria included the use of alcohol or illicit drugs (determined by urinalysis), age under 18 years, or intention to formula feed. Patients who agreed to participate in the study signed an informed consent form after receiving a detailed explanation of the nature and objectives of the study.

This study was approved on August 10, 2021, by the Ethics Committee of the Vall d’Hebron Research Institute (project number: 558/2020) and was funded by the National Plan on Drugs of the Health Ministry of the Government of Spain (grant number: 2021/047).

### 2.2 Assessment

An *ad hoc* designed questionnaire was used to collect baseline sociodemographic and clinical characteristics at the time of the enrolment. Clinical data included psychiatric history, obstetric information, and relevant medical history. Relevant medical history was defined as chronic or clinically relevant medical conditions recorded in the maternal clinical history, including endocrine, cardiovascular, respiratory, infectious, neurological, renal, hepatic, autoimmune, or other clinically relevant disorders. Additional clinical data, including height (m) and weight (kg), were obtained by physical examination at the time of admission. Sociodemographic features recorded were nationality, educational level, civil status, and employment. Psychiatric data included cannabis use information (onset of cannabis use, years of use, way of administration, amount of cannabis used during previous and during pregnancy), psychiatric disorders (according to clinical records and following DSM-5 criteria) and prior follow-up by mental health services. Obstetric and neonatal information included birth route, sex, gestational age at birth (weeks), birth weight (g), birth height and head circumference, obstetric history, previous miscarriage or abortion and need for admission to intensive care unit. Birth weight and birth length percentiles were calculated using World Health Organization (WHO) growth references and were adjusted for gestational age and sex.

### 2.3 Sample collection and analytical procedure

Participants were asked to provide a kit of samples of urine (10 mL), saliva (5 mL) and breast milk (5 mL) extracted at the same time in three consecutive stages:

• The 1st stage in the first 24 hours after delivery.
• The 2nd stage in the first 48 hours after delivery.
• The 3rd stage, approximately one week after delivery (7 days ± 48 hours).

The exact time of each sample collection was recorded for subsequent analysis. Although sample collection was scheduled at predefined postpartum time points, some samples were collected slightly before or after the target time because of logistical constraints, including weekend scheduling. Urine, saliva, and breast milk samples belonging to the same collection kit were obtained at the same time whenever possible. All samples were collected at Vall d’Hebron University Hospital by trained research and nursing staff according to standardized procedures.

Urine samples were collected by spontaneous voiding into a sterile polypropylene urine collection container. Saliva samples were collected by passive drool into a sterile polypropylene collection tube, without the use of swabs or absorbent devices. Participants were instructed to allow saliva to accumulate in the mouth and then expectorate directly into the collection tube until approximately 5 mL of saliva was obtained. Breast milk samples were obtained by manual full expression performed by the participant herself under the guidance of trained nursing staff. In this context, “full expression” referred to collecting all milk available at the time of sampling, which during early lactation generally corresponded to small colostrum volumes rather than to a predetermined large volume. Milk was collected from both breasts whenever possible into a sterile polypropylene collection tube. When milk was obtained from both breasts, the samples were pooled and gently mixed before aliquoting to ensure a homogeneous sample for analysis. A 5 mL aliquot was then transferred for laboratory analysis; when less than 5 mL was available, the maximum available volume was transferred and processed. Samples collected after participants reported cannabis use between sampling stages were not included in the abstinent group analyses and were presented separately.

Breast milk, urine, and saliva samples were received at the Core Laboratory where they were stored at a temperature of 4–6 °C until shipment to the reference laboratory. Samples were transported on the same day under refrigerated conditions. Preparation of samples was performed following the specific inserts of the reference laboratory. Subsequently, samples were analyzed by liquid chromatography-tandem mass spectrometry (LC-MS/MS) using a 1290 liquid chromatography system coupled to a 6430 triple quadrupole tandem mass spectrometer (QQQ-MS/MS) (Agilent^®^) (29, 30). Chromatographic separation was performed on a reverse-phase C18 column under gradient elution using a mobile phase composed of water with 0.1% formic acid and methanol with 0.1% formic acid at a flow rate of 0.35 mL/min. The column temperature was 35°C, and a total analytical run time was 15 minutes. In breast milk and saliva samples, the target analyte was Δ9-tetrahydrocannabinol (Δ9-THC), whereas urine samples were analyzed for 11-nor-9-carboxy-Δ9-tetrahydrocannabinol (THC-COOH). The limit of quantification (LOQ) for breast milk and saliva samples was established at 5 ng/mL, whereas for urine samples it was 2 ng/mL. The LOQ was determined by analyzing 10 blank replicates and calculating ten times the signal intensity of the blank sample with the highest response.

### 2.4 Statistical analysis

An initial descriptive analysis was performed, including means, standard deviations, and percentages. Subsequently, bivariate analysis was performed using non-parametric tests due to the limited sample size. Spearman’s rank correlation coefficient was calculated to assess correlations between Δ9-THC concentrations in breast milk and saliva and urinary concentrations of THC-COOH and selected quantitative variables relevant to the study objectives, including maternal age, body mass index (BMI), and self-reported cannabis use during pregnancy expressed as joints per day. As Δ9-THC concentrations in breast milk samples collected one week after delivery were below the LOQ, comparative analyses evaluating changes over time and their statistical significance could not be performed for this matrix. Statistical significance was assessed using a two-tailed hypothesis, with p-values <0.05 considered statistically significant. All analyses were performed using SPSS Statistics version 22.0 (IBM Corp., Armonk, NY, USA).

## 3 Results

The initial sample comprised 15 participants. However, two participants were excluded from the final analysis for different reasons: one was identified as a false positive during childbirth screening confirmed by quantitative urinalysis, while the other one provided the samples two weeks after delivery, outside the established period. Therefore, the final analyzed sample consisted of 13 participants.

**Figure 1:**
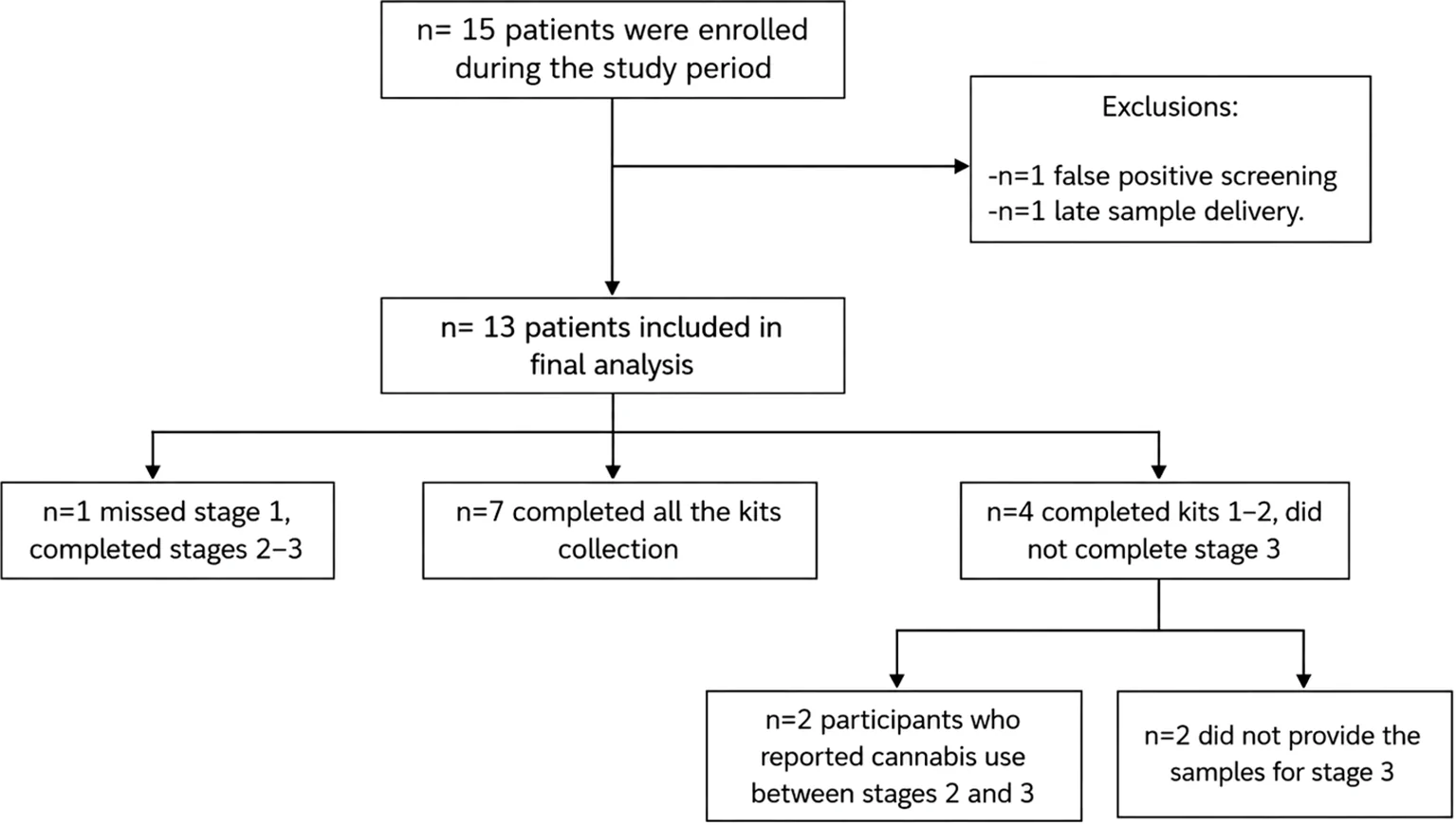
Flow chart of participants included.

Among them, seven participants completed the full collection of samples across the three study stages. One participant missed the first stage collection but provided samples for stages 2 and 3. In addition, five participants completed sample collection for the first two stages but did not complete stage 3 (three were lost to follow up before the third stage one week later, and two reported cannabis use between stages 2 and 3) (**Figure 1**).

The sociodemographic and clinical characteristics are presented in **Table 1**. Mean maternal age was 28.0 ± 6.77 years and mean gestational age at birth 38.33 ± 1.51 weeks. Interestingly, regarding cannabis use history, the women reported using more than six joints per day before pregnancy, whereas during pregnancy, this consumption decreased to 1.45 ± 0.93 joints per day.

**Table 1:** Baseline sociodemographic and clinical characteristics.

Variable	Remained abstinent after delivery(n=11)	Reported cannabis use after delivery(n=2)	Total sample(n=13)
Maternal sociodemographic and clinical characteristics			
Age (years), mean ± SD	28.00 ± 6.77	30.00 ± 2.83	28.31 ± 6.28
Height (cm), mean ± SD	161.91 ± 6.55	159.50 ± 7.78	161.54 ± 6.45
Weight (kg), mean ± SD	66.88 ± 14.57	43.75 ± 8.84	63.32 ± 16.09
Body mass index, mean ± SD	25.52 ± 5.55	17.00 ± 1.98	24.21 ± 6.02
Nationality: Spanish, n (%)	9 (81.8%)	2 (100.0%)	11 (84.6%)
Nationality: Other, n (%)	2 (18.2%)	0 (0.0%)	2 (15.4%)
Civil status: Single, n (%)	2 (18.2%)	1 (50.0%)	3 (23.1%)
Civil status: Married/partner, n (%)	9 (81.8%)	1 (50.0%)	10 (76.9%)
Education duration <6 years, n (%)	6 (54.5%)	2 (100.0%)	8 (61.5%)
Education duration >6 years, n (%)	5 (45.5%)	0 (0.0%)	5 (38.5%)
Employment status: Employed, n (%)	3 (27.3%)	1 (50.0%)	4 (30.8%)
Employment status: Unemployed, n (%)	8 (72.7%)	1 (50.0%)	9 (69.2%)
Relevant medical history: Yes, n (%)	2 (18.2%)	1 (50.0%)	3 (23.1%)
Relevant medical history: No, n (%)	9 (81.8%)	1 (50.0%)	10 (76.9%)
Pregnancy, delivery, and neonatal outcomes			
Gestational age at delivery (weeks), mean ± SD	38.33 ± 1.51	34.20 ± 0.28	37.70 ± 2.08
Type of delivery: Vaginal delivery, n (%)	6 (54.5%)	0 (0.0%)	6 (46.2%)
Type of delivery: Assisted vaginal delivery, n (%)	1 (9.1%)	0 (0.0%)	1 (7.7%)
Type of delivery: Cesarean section, n (%)	4 (36.4%)	2 (100.0%)	6 (46.2%)
THC in newborn urine: Positive, n (%)	6 (54.5%)	0 (0.0%)	6 (46.2%)
THC in newborn urine: Negative, n (%)	5 (45.5%)	2 (100.0%)	7 (53.8%)
Birth weight (g), mean ± SD	2745.0 ± 409.41	2030.0 ± 70.71	2635.0 ± 460.65
Birth weight percentile, mean ± SD	21.27 ± 16.19	29.50 ± 2.12	22.54 ± 15.11
Newborn length at birth (cm), mean ± SD	48.19 ± 1.83	42.0 ± 1.41	47.23 ± 2.89
Birth length percentile, mean ± SD	31.36 ± 25.50	13.50 ± 14.85	28.62 ± 24.60
Head circumference (cm), mean ± SD	33.64 ± 1.80	30.50 ± 0.71	33.15 ± 2.04
Head circumference percentile, mean ± SD	33.82 ± 26.27	24.0 ± 7.07	32.31 ± 25.35
Apgar score at 1 min, mean ± SD	8.09 ± 2.55	6.50 ± 0.71	7.85 ± 2.41
Psychiatric and substance use history			
Family history of psychiatric disorders: Yes, n (%)	1 (9.1%)	2 (100.0%)	3 (23.1%)
Family history of psychiatric disorders: No, n (%)	10 (90.9%)	0 (0.0%)	10 (76.9%)
Personal history of psychiatric disorders: Yes, n (%)	5 (45.5%)	1 (50.0%)	6 (46.2%)
Personal history of psychiatric disorders: No, n (%)	6 (54.5%)	1 (50.0%)	7 (53.8%)
Tobacco use disorder: Yes, n (%)	6 (54.5%)	2 (100.0%)	8 (61.5%)
Tobacco use disorder: No, n (%)	5 (45.5%)	0 (0.0%)	5 (38.5%)
Cannabis use onset (years), mean ± SD	15.45 ± 2.11	16.00 ± 0.00	15.54 ± 1.94
Cannabis use before pregnancy (joints/day), mean ± SD	6.27 ± 6.34	15.5 ± 20.51	7.69 ± 8.98
Cannabis use during pregnancy (joints/day), mean ± SD	1.45 ± 0.93	1.0 ± 0.0	1.39 ± 0.87

Data are presented as mean ± standard deviation or n (%).

BMI, body mass index; SD, standard deviation; THC, tetrahydrocannabinol.

Education duration refers to total years of formal education. Relevant medical history refers to chronic or clinically relevant medical conditions recorded in the maternal clinical history.

Birth weight and birth length percentiles were calculated using World Health Organization (WHO) growth references and were adjusted for gestational age and sex.

### 3.1 Evolution of Δ9-THC concentrations in breast milk and saliva, and THC-COOH concentrations in urine across the three study stages

The cannabinoid concentrations obtained in our study across the three sampling stages are summarized in **Table 2**. Among women who maintained abstinence during the first postpartum week, urinary concentrations of THC-COOH showed a progressive decline over time but remained quantifiable throughout all study stages (100% at stage 1, 90.91% at stage 2, and 87.5% at stage 3). In contrast, Δ9-THC concentrations in breast milk progressively decreased during the study period, with quantifiable levels observed in 40% of samples at stage 1, 27.28% at stage 2, and 0% at stage 3.

**Table 2:** Salivary and breast milk Δ9-THC concentrations and urinary THC-COOH concentrations across postpartum sampling stages.

	Stage 1 (24h)			Stage 2 (48h)			Stage 3 (1 week)		
Participant	Urine (ng/mL)	Saliva (ng/mL)	Breast milk (ng/mL)	Urine (ng/mL)	Saliva (ng/mL)	Breast milk (ng/mL)	Urine (ng/mL)	Saliva (ng/mL)	Breast milk (ng/mL)
Participants who remained abstinent									
1^(1)^	1586	27	40	1002	13	49	NC	NC	NC
2	36	<LOQ	<LOQ	<LOQ	<LOQ	<LOQ	11	<LOQ	<LOQ
3	21	<LOQ	<LOQ	18	<LOQ	<LOQ	43	<LOQ	<LOQ
4	33	<LOQ	<LOQ	11	<LOQ	<LOQ	<LOQ	<LOQ	<LOQ
5^(1)^	358	<LOQ	34	224	<LOQ	81	NC	NC	NC
6	571	<LOQ	<LOQ	152	<LOQ	<LOQ	151	<LOQ	<LOQ
7^(2)^	NC	NC	NC	100	<LOQ	<LOQ	53	<LOQ	<LOQ
8^(1)^	1506	<LOQ	24	862	<LOQ	19	NC	NC	NC
9	186	<LOQ	5	209	<LOQ	<LOQ	78	<LOQ	<LOQ
10	35	<LOQ	<LOQ	69	<LOQ	<LOQ	17	<LOQ	<LOQ
11	20	<LOQ	<LOQ	17	<LOQ	<LOQ	20	<LOQ	<LOQ
Quantifiable samples, n/N (%)	10/10	1/10	4/10	10/11	1/11	3/11	7/8	0/8	0/8
	(100%)	(10%)	(40%)	(90.91%)	(9.09%)	(27.28%)	(87.5%)	(0%)	(0%)
Participants who reported cannabis use between stages 2 and 3									
12^(3)^	31	<LOQ	<LOQ	74	<LOQ	8	407	108	13
13^(3)^	78	<LOQ	<LOQ	263	<LOQ	<LOQ	NC	NC	NC

^(1)^ Participants did not provide samples for stage 3; ^(2)^ Participant who lost stage 1 but provided samples for stage 2 and 3; ^(3)^ Participants who reported cannabis use between stages 2 and 3. <LOQ, below the limit of quantification; NC, not collected or not included in the final analysis because the sample kit was incomplete.

**Figure 2:**
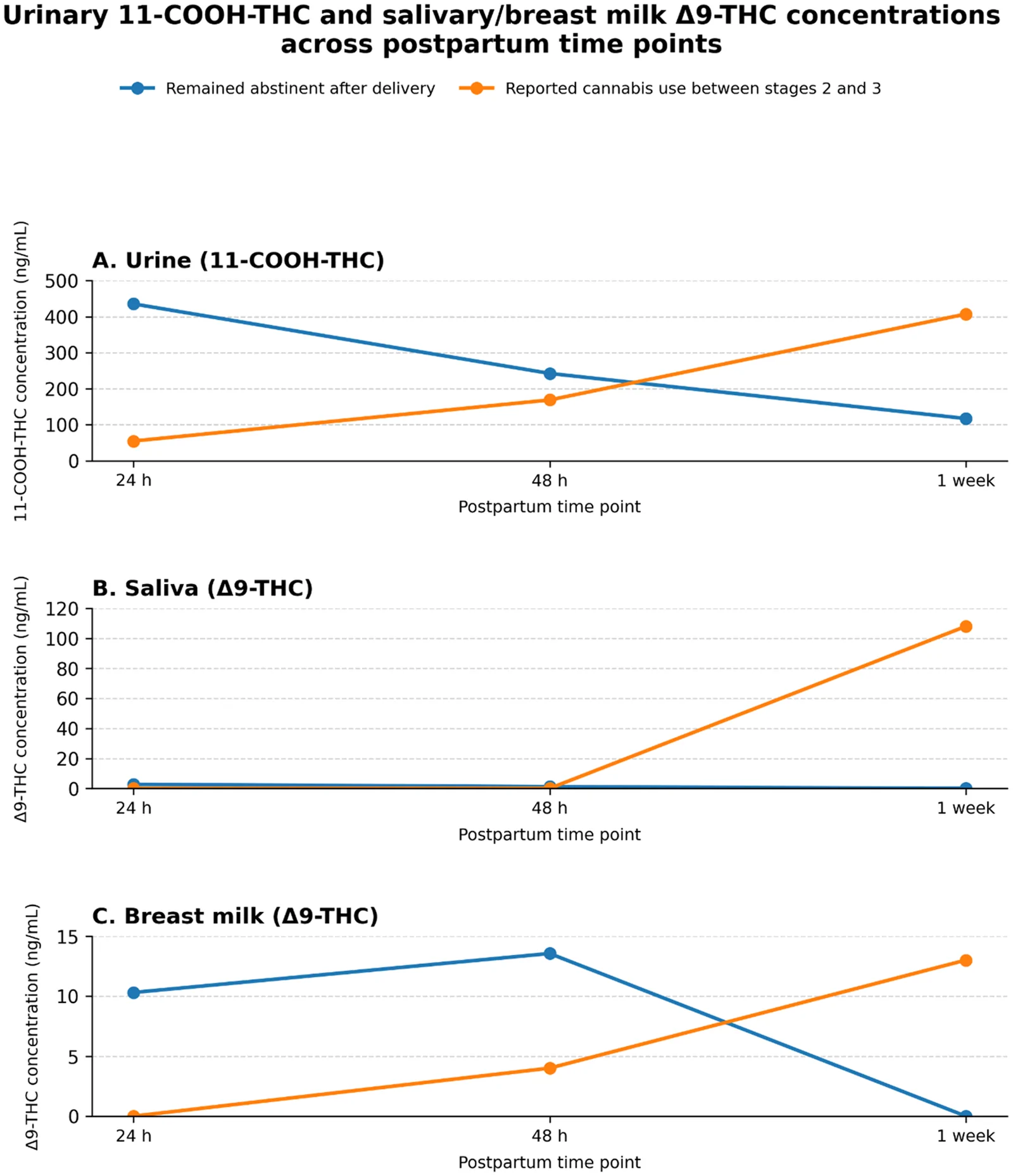
Mean concentrations of urinary 11-nor-9-carboxy-Δ9-tetrahydrocannabinol (11-COOH-THC) and salivary/breast milk Δ9-tetrahydrocannabinol (Δ9-THC) measured at 24h, 48h, and 1 week postpartum according to cannabis use status after delivery. Panel **(A)** shows urinary 11-COOH-THC concentrations, Panel **(B)** shows salivary Δ9-THC concentrations, and Panel **(C)** shows breast milk Δ9-THC concentrations. Lines represent mean concentrations (ng/mL).Values below the limit of quantification (<LOQ) are displayed as 0 for graphical purposes only and should not be interpreted as true zero concentrations. Sample sizes were as follows: abstinent group, n = 10, 11, and 8 at 24h, 48h, and 1 week respectively; participants who reported cannabis use between stages 2 and 3, n = 2 at 24h and 48h, and n = 1 at 1 week.

Salivary Δ9-THC concentrations were generally low across the study period and were frequently below the limit of quantification. In most cases, Δ9-THC concentrations in saliva were lower than those detected in breast milk samples collected at the same sampling time points. Notably, our findings showed a transient increase in breast milk Δ9-THC concentrations at 48 hours postpartum (**Table 2**, **Figure 2**).

Correlations between cannabinoid concentrations in the different biological matrices and selected clinical variables including maternal age, body mass index (BMI), and number of joints used during pregnancy, are shown in **Table 3**. Correlations analyses across all three study stages could only be performed for urinary THC-COOH concentrations. For saliva and breast milk, analyses were restricted to stages 1 and 2 because Δ9-THC concentrations were below the LOQ at stage 3. Consequently, these variables showed no variability at the third sampling time point and were therefore not suitable for Spearman’s rank correlation analysis.

**Table 3:** Spearman correlation matrix between maternal clinical variables, self-reported cannabis use during pregnancy, urinary THC-COOH concentrations, and salivary/breast milk Δ9-THC concentrations across postpartum sampling stages.

	1	2	3	4	5	6	7	8	9	10	11	12
1. Age	–											
2. BMI	0.278	–										
3. Cannabis use during pregnancy (joints/day)	-0.269	-.657*	–									
4. Urine THC (T1)	-0.006	-0.189	-0.017	–								
5. Saliva THC (T1)	-0.443	-0.519	0.218	.660*	–							
6. Breast milk THC (T1)	-0.378	-0.387	-0.009	.770**	.653*	–						
7. Urine THC (T2)	-0.042	-0.215	-0.017	.978**	.714*	.815**	–					
8. Saliva THC (T2)	-0.392	-0.425	0.194	.660*	1.000**	.653*	.717*	–				
9. Breast milk THC (T2)	-0.376	-0.202	-0.074	0.455	0.426	.900**	0.501	0.435	–			
10. Urine THC (T3)	0.488	0.149	-0.163	.985**	.a	.967**	.985**	.a	.977**	–		
11. Saliva THC (T3)	.a	.a	.a	.a	.a	.a	.a	.a	.a	.a	–	
12. Breast milk THC (T3)	.a	.a	.a	.a	.a	.a	.a	.a	.a	.a	.a	–

*p < 0.05; **p < 0.01; a. The statistic could not be computed because at least one of the variables is constant.

BMI, Body Mass Index; T1, first sample collection; T2, second sample collection; T3, third sample collection.

None of the clinical variables evaluated, including maternal age, BMI, or number of joints used during pregnancy, showed significant correlations with THC-COOH concentrations in urine and Δ9-THC concentrations in saliva or milk. Urinary THC-COOH concentrations at stage 1 were significantly correlated with Δ9-THC concentrations in saliva and milk. Breast milk Δ9-THC concentrations at stage 1 were also significantly correlated with Δ9-THC concentrations in saliva and urinary THC-COOH concentrations across the different stages. In contrast, breast milk Δ9-THC concentrations at stage 2 were not significantly correlated with saliva Δ9-THC or urinary THC-COOH concentrations, although they were significantly correlated with breast milk Δ9-THC concentrations at other sampling time points (p<0.05).

## 4 Discussion

This study provides clinically relevant findings at a crucial time point in maternal and child health. The main finding is that, among participants who discontinued cannabis use immediately after delivery, Δ9-THC concentrations in breast milk were no longer quantifiable within the first postpartum week. This finding may contribute to future discussions regarding breastfeeding management among women with recent cannabis exposure. However, given the preliminary nature of these results and the small sample size, they should not be interpreted as providing direct clinical guidance.

Although a larger sample size was originally planned, recruitment proved challenging for several reasons. One of these main reasons is the improvement in the healthcare network for substance use in the perinatal period in recent years (e.g. women are screened for substance use during pregnancy and referred to a perinatal mental health specialist, which may contribute to higher cannabis discontinuation rates and, consequently, to fewer positive drug tests at delivery) (18, 31). In addition, the implementation of strategies to mitigate the risk of cannabis use during pregnancy and breastfeeding may also have influenced recruitment (32). Similar recruitment difficulties have been reported in previous studies conducted in this field (24, 27).

In the present study, THC-COOH remained quantifiable in urine for longer than Δ9-THC in breast milk and saliva among participants who discontinued cannabis use after delivery. None of the participants who completed all three sampling stages and remained abstinent showed quantifiable Δ9-THC concentrations in breast milk one week postpartum, whereas THC-COOH remained quantifiable in urine. These findings suggest that urine testing, which commonly detects THC-COOH, may not be an appropriate biological marker for estimating the presence or absence of Δ9-THC in breast milk among women who have discontinued cannabis use. This distinction is clinically relevant, as urine tests are widely used in clinical settings but may reflect prior cannabis exposure rather than current Δ9-THC concentrations in breast milk.

These findings are partially consistent with previous research suggesting that Δ9-THC concentrations in breast milk may decline within a few days after cannabis cessation (27). However, they should be interpreted cautiously, as another study reported quantifiable Δ9-THC concentrations in breast milk up to six weeks after cessation and suggested that concentrations may increase during the first two weeks postpartum (33). These divergent findings may be explained by several factors, differences in cannabis use patterns, timing since last use, frequency and route of administration, analytical methods, and postpartum changes in breast milk composition.

Changes in milk composition during the first days after delivery may also contribute to variability in measured Δ9-THC concentrations. Colostrum, which is produced during the first few days postpartum, is rich in protein, antibodies, and other immune factors but contains lower fat and sugar concentrations than mature milk (34, 35). As lactation progresses, breast milk composition changes and mature milk becomes more variable in fat content. Differences between foremilk, which is produced at the beginning of a feeding and is generally lower in fat, and hindmilk, which is produced later and is richer in fat, may influence Δ9-THC concentrations because it is highly lipophilic. Therefore, variation in the timing of sample collection in relation to feeding may have contributed to variability in breast milk Δ9-THC concentrations.

In addition, all breast milk samples from mothers whose newborns had a negative qualitative urine test for cannabis exposure at birth, were below the LOQ. Although this observation may be clinically relevant, it should be interpreted with caution due to the small sample size and the exploratory nature of the study. Future research should evaluate whether neonatal toxicology results could contribute to risk stratification or breastfeeding counseling in women with recent cannabis exposure.

Frequency of cannabis use has been recognized as an important factor associated with the presence of Δ9-THC in breast milk (25, 26). However, in the present study, no significant correlations were found between maternal BMI, self-reported cannabis use during pregnancy and Δ9-THC concentration in breast milk. This may be explained by the small sample size, limited statistical power, and the relatively low reported cannabis use during pregnancy, with most participants reporting a reduction to approximately one joint per day.

The present study should be evaluated regarding its multiple limitations and bias. First, the sample size was small, mainly due to recruitment challenges. Although this limits the generalizability of the findings, similar studies in this field have also reported small sample sizes and recruitment difficulties (24, 25, 33). Second, plasma Δ9-THC concentrations were not measured. However, because oral fluid has a detection window that may be closer to blood than urine (36), saliva was selected as a non-invasive biological matrix. Third, although most participants reported abstinence from cannabis use after delivery, the exact timing of last cannabis use before delivery could not be precisely determined. This is particularly relevant because cannabis products may vary substantially in Δ9-THC concentration, route of administration, and frequency of use.

Another limitation is that the timing of breast milk collection in relation to feeding was not systematically recorded. Time since last feeding is one of the factors associated with milk fat concentration (37). These changes in composition may cause the results to be inconsistent due to the higher or lower concentration of fat in the different expressions of breast milk. Nevertheless, recent studies found that the qualitative lipid profile (the proportion of specific lipid species) remains remarkably stable throughout a single breastfeeding session for each individual mother indicating that the lipid profile of breast milk is a stable individual characteristic that reflects the particularities of each mother (38).

Despite these limitations, this study has several strengths. To our knowledge, few studies have simultaneously assessed cannabinoid related measures in breast milk, saliva, and urine during the immediate postpartum period. The longitudinal design, the collection of paired biological samples at predefined postpartum time points, and the use of LC-MS/MS provide valuable preliminary data in a limited and clinically relevant field.

In conclusion, this study suggests that Δ9-THC levels in breast milk may decline rapidly after postpartum cannabis cessation, becoming non-quantifiable within the first week among women who discontinued use at delivery. In contrast, THC-COOH remained quantifiable in urine for a longer period, indicating that urine testing may primarily reflect previous cannabis exposure rather than current Δ9-THC concentrations in breast milk. In our study, salivary Δ9-THC measurements showed limited concordance with breast milk Δ9-THC concentrations and should therefore be interpreted cautiously as a potential surrogate marker.

Although the small sample size and methodological limitations warrant cautious interpretation, these findings contribute valuable evidence to a limited and evolving field. These preliminary findings highlight the importance of considering biological matrix selection, timing of sample collection, and postpartum milk composition when evaluating cannabis exposure during lactation. Larger prospective studies are needed to confirm these findings and to establish evidence-based guidelines that support safe breastfeeding practices in women with prior cannabis use.

## Data Availability

The original contributions presented in the study are included in the article/supplementary material. Further inquiries can be directed to the corresponding author.
