# Correction: Breast milk Δ9-tetrahydrocannabinol in cannabis users during the postpartum period: correlation between breast milk, maternal urine and saliva samples during early lactation

**DOI:** 10.3389/fpsyt.2026.1945694

**Published:** 2026-08-10

**Authors:** Miguel Sandonís, Maia Brik, Clara Ramirez, Laura Castellote, Anna Creus, Gemma Parramón, Anna Suy, Josep Antoni Ramos-Quiroga, Raul Felipe Palma-Álvarez, Constanza Daigre

**Affiliations:** 1 Department of Mental Health, Hospital Universitari Vall d’Hebron, Barcelona, Spain; 2 Psychiatry, Mental Health and Addictions Group, Vall d’Hebron Research Institute (VHIR), Instituto de Investigación Sanitaria Hospital Universitari Vall d’Hebron (IIS IR-HUVH), Barcelona, Catalonia, Spain; 3 Biomedical Research Networking Center in Mental Health (CIBERSAM), Barcelona, Spain; 4 Department of Psychiatry and Forensic Medicine, Universitat Autònoma de Barcelona, Barcelona, Spain; 5 Department of Obstetrics and Gynecology, Universitat Autònoma de Barcelona, Barcelona, Spain; 6 Obstetrics Department, Maternal-Fetal Medicine Unit, Hospital Universitari Vall d’Hebron, Barcelona, Spain; 7 Clinical Biochemistry Research Group, Vall d’Hebron Research Institute (VHIR), Biochemical Core Facilities, Instituto de Investigación Sanitaria Hospital Universitari Vall d’Hebron (IIS IR-HUVH), Barcelona, Catalonia, Spain; 8 Department of Neonatology, Hospital Universitari Vall d’Hebron, Barcelona, Spain

**Keywords:** cannabis, pregnancy, breastfeeding, postpartum, perinatal

In the published article, in Section 3, Results, page 5, **Table 1**, the neonatal urine toxicology data were reported incorrectly.

**Table 1:** Baseline sociodemographic and clinical characteristics.

Variable	Remained abstinent after delivery (n=11)	Reported cannabis use after delivery (n=2)	Total sample (n=13)
Maternal sociodemographic and clinical characteristics			
Age (years), mean ± SD	28.00 ± 6.77	30.00 ± 2.83	28.31 ± 6.28
Height (cm), mean ± SD	161.91 ± 6.55	159.50 ± 7.78	161.54 ± 6.45
Weight (kg), mean ± SD	66.88 ± 14.57	43.75 ± 8.84	63.32 ± 16.09
Body mass index, mean ± SD	25.52 ± 5.55	17.00 ± 1.98	24.21 ± 6.02
Nationality: Spanish, n (%)	9 (81.8%)	2 (100.0%)	11 (84.6%)
Nationality: Other, n (%)	2 (18.2%)	0 (0.0%)	2 (15.4%)
Civil status: Single, n (%)	2 (18.2%)	1 (50.0%)	3 (23.1%)
Civil status: Married/partner, n (%)	9 (81.8%)	1 (50.0%)	10 (76.9%)
Education duration <6 years, n (%)	6 (54.5%)	2 (100.0%)	8 (61.5%)
Education duration >6 years, n (%)	5 (45.5%)	0 (0.0%)	5 (38.5%)
Employment status: Employed, n (%)	3 (27.3%)	1 (50.0%)	4 (30.8%)
Employment status: Unemployed, n (%)	8 (72.7%)	1 (50.0%)	9 (69.2%)
Relevant medical history: Yes, n (%)	2 (18.2%)	1 (50.0%)	3 (23.1%)
Relevant medical history: No, n (%)	9 (81.8%)	1 (50.0%)	10 (76.9%)
Pregnancy, delivery, and neonatal outcomes			
Gestational age at delivery (weeks), mean ± SD	38.33 ± 1.51	34.20 ± 0.28	37.70 ± 2.08
Type of delivery: Vaginal delivery, n (%)	6 (54.5%)	0 (0.0%)	6 (46.2%)
Type of delivery: Assisted vaginal delivery, n (%)	1 (9.1%)	0 (0.0%)	1 (7.7%)
Type of delivery: Cesarean section, n (%)	4 (36.4%)	2 (100.0%)	6 (46.2%)
THC in newborn urine: Positive, n (%)	6 (54.5%)	0 (0.0%)	6 (46.2%)
THC in newborn urine: Negative, n (%)	5 (45.5%)	2 (100.0%)	7 (53.8%)
Birth weight (g), mean ± SD	2745.0 ± 409.41	2030.0 ± 70.71	2635.0 ± 460.65
Birth weight percentile, mean ± SD	21.27 ± 16.19	29.50 ± 2.12	22.54 ± 15.11
Newborn length at birth (cm), mean ± SD	48.19 ± 1.83	42.0 ± 1.41	47.23 ± 2.89
Birth length percentile, mean ± SD	31.36 ± 25.50	13.50 ± 14.85	28.62 ± 24.60
Head circumference (cm), mean ± SD	33.64 ± 1.80	30.50 ± 0.71	33.15 ± 2.04
Head circumference percentile, mean ± SD	33.82 ± 26.27	24.0 ± 7.07	32.31 ± 25.35
Apgar score at 1 min, mean ± SD	8.09 ± 2.55	6.50 ± 0.71	7.85 ± 2.41
Psychiatric and substance use history			
Family history of psychiatric disorders: Yes, n (%)	1 (9.1%)	2 (100.0%)	3 (23.1%)
Family history of psychiatric disorders: No, n (%)	10 (90.9%)	0 (0.0%)	10 (76.9%)
Personal history of psychiatric disorders: Yes, n (%)	5 (45.5%)	1 (50.0%)	6 (46.2%)
Personal history of psychiatric disorders: No, n (%)	6 (54.5%)	1 (50.0%)	7 (53.8%)
Tobacco use disorder: Yes, n (%)	6 (54.5%)	2 (100.0%)	8 (61.5%)
Tobacco use disorder: No, n (%)	5 (45.5%)	0 (0.0%)	5 (38.5%)
Cannabis use onset (years), mean ± SD	15.45 ± 2.11	16.00 ± 0.00	15.54 ± 1.94
Cannabis use before pregnancy (joints/day), mean ± SD	6.27 ± 6.34	15.5 ± 20.51	7.69 ± 8.98
Cannabis use during pregnancy (joints/day), mean ± SD	1.45 ± 0.93	1.0 ± 0.0	1.39 ± 0.87

Data are presented as mean ± standard deviation or n (%).

BMI, body mass index; SD, standard deviation; THC, tetrahydrocannabinol.

Education duration refers to total years of formal education. Relevant medical history refers to chronic or clinically relevant medical conditions recorded in the maternal clinical history.

Birth weight and birth length percentiles were calculated using World Health Organization (WHO) growth references and were adjusted for gestational age and sex.

The published table included only the row “THC in newborn urine: Negative, n (%)”, and the values shown in that row were incorrect. The table should include both neonatal urine toxicology categories, positive and negative.

The corrected values are shown in the table below. Specifically, 6/13 newborns had a positive urine THC result and 7/13 newborns had a negative urine THC result. This correction affects only the descriptive neonatal toxicology data presented in **Table 1** and does not affect the study results, interpretation, or conclusions.

The corrected **Table 1** is below.

The original version of this article has been updated.

